# Supplementary material for: Cartilage Homeostasis Affects Femoral Head Necrosis Induced by Methylprednisolone in Broilers
Source: Int J Mol Sci. 2020 Jul 8;21(14):4841. doi: 10.3390/ijms21144841 (PMC7402315; doi:10.3390/ijms21144841)
Supplement: Supplementary file 1 [file ijms-21-04841-s001.pdf]

International Journal of *Molecular Sciences*

MDPI

Supplementary

Table S1. Sequences of primers used to amplify specific mRNAs by qRT-PCR.

Target gene	Primer sequence (5'-3')
GAPDH	Forward: GAACATCATCCCAGCGTCCA<br>Reverse: CGGCAGGTCAGGTCAACAAC
collagen-2	Forward: ACCTACAGCGTCTTGGAGGA<br>Reverse: ATATCCACGCCAAACTCCTG
collagen-10	Forward: GCCTTCCAGGTCAGCCAGGTAT<br>Reverse: TTGCCGATGCCAACTTCTCCAG
aggrecan	Forward: TGCAAGGCAAAGTCTTCTACG<br>Reverse: GGCAGGGTTCAGGTAAACG
β-catenin	Forward: GGTGGCATCGAGGCTCTTGTG<br>Reverse: GCTTCCTGATGTCTGCTGGTGAG
LRP-5	Forward: GCAGCCACATGACCTCAGCATT<br>Reverse: ACCTCGCAGCACCATCCCTATT
LRP-6	Forward: TCACGGCAGAACATCATTCGGAAG<br>Reverse: CGGCTGTAGATGTCAATGCTCAGG
HIF-1α	Forward: CAGCCAGGTGCCGAAGAAGC<br>Reverse: ATGGTCAGCCTCATAATGGATGCC
HIF-2α	Forward: CTGTTGACGATGAGCAGTGCCT<br>Reverse: CCAGGTGTTGGAGCCAGTTGTG
VEGFA	Forward: GAGTTGTCGAAGGCTGCTCC<br>Reverse: GCAACCCGCACATCTCATCA
Bcl-2	Forward: CACCTGGATGACCGAGTACC<br>Reverse: GTCCAAGATAAGCGCCAAGA
caspase-3	Forward: TGGCGATGAAGGACTCTTCT<br>Reverse: TCATCTGGTCCACTGTCTGC
Bid	Forward: GCCTGACCCTGAGGTAAATG<br>Reverse: ACAGGCACCGTGTTATCTCC
LC3-I	Forward: GCTGCCAGTGCTGGACAAGAC<br>Reverse: TCCTCATCCTTCTCCTGCTCGTAG
LC3-II	Forward: CCTGGTGCCAGATCACGTCAAC<br>Reverse: AAGCCGTCCTCGTCCTTCTCG
Beclin-1	Forward: ACCGCAAGATTGTGGCTGAAGAC<br>Reverse: TGAGCATAACGCATCTGGTTCTCC
PCNA	Forward: GCCATGGGCGTCAACCTAAA<br>Reverse: AGCCAACGTATCCGCATTGT
KI-67	Forward: AAAAACCTGATTCCTGAACAATCTG<br>Reverse: GACCTAGAGCTATCAGGCTGTGAAG
TNF-α	Forward: CTCAGGACAGCCTATGCCAACAAG<br>Reverse: GCCACCACACGACAGCCAAG
IL-1β	Forward: GGTCAACATCGCCACCTACA<br>Reverse: CATACGAGATGCAAACCAGCAA
IGF-1	Forward: GTATGTGGAGACAGAGGCTTC<br>Reverse: TTTGGCATATCAGTGTGGCGC
MMP-9	Forward: GCCATCACTGAGATCAATGGAG<br>Reverse: GATAGAGAAGGCGCCCTGAGT
CDMP-1	Forward: CGCCTCCAACTTGCTGTGTCC<br>Reverse: TCCACCACCATGTCCTCGTACTG
BMP-3	Forward: CTGACATTGGCTGGAGCGAGTG<br>Reverse: TCTGGATGGTGGCATGGTTGGA

© 2020 by the authors. Submitted for possible open access publication under the terms and conditions of the Creative Commons Attribution (CC BY) license (http://creativecommons.org/licenses/by/4.0/).
